# *FAM20A* Gene Mutation: Amelogenesis or Ectopic Mineralization?

**DOI:** 10.3389/fphys.2017.00267

**Published:** 2017-05-03

**Authors:** Guilhem Lignon, Fleur Beres, Mickael Quentric, Stephan Rouzière, Raphael Weil, Muriel De La Dure-Molla, Adrien Naveau, Renata Kozyraki, Arnaud Dessombz, Ariane Berdal

**Affiliations:** ^1^Molecular Oral Pathophysiology, Cordeliers Research Center, UMRS 1138 Institut National de la Santé et de la Recherche Médicale, Paris-Descartes, Pierre-et-Marie-Curie, Paris-Diderot UniversitiesParis, France; ^2^Laboratoire de Physique des Solides, Centre National de la Recherche Scientifique, Univ. Paris-Sud, Université Paris-SaclayOrsay Cedex, France; ^3^Reference Center of Rare Buccal and Facial Malformations MAFACE-Rothschild Hospital, APHPParis, France

**Keywords:** amelogenesis imperfecta, *FAM20A*, rare disease, mineral, matrix biology

## Abstract

**Background and objective:** *FAM20A* gene mutations result in enamel renal syndrome (ERS) associated with amelogenesis imperfecta (AI), nephrocalcinosis, gingival fibromatosis, and impaired tooth eruption. FAM20A would control the phosphorylation of enamel peptides and thus enamel mineralization. Here, we characterized the structure and chemical composition of unerupted tooth enamel from ERS patients and healthy subjects.

**Methods:** Tooth sections were analyzed by Scanning Electron Microscopy (SEM), Energy Dispersive Spectroscopy (EDS), X-Ray Diffraction (XRD), and X-Ray Fluorescence (XRF).

**Results:** SEM revealed that prisms were restricted to the inner-most enamel zones. The bulk of the mineralized matter covering the crown was formed by layers with varying electron-densities organized into lamellae and micronodules. Tissue porosity progressively increased at the periphery, ending with loose and unfused nanonodules also observed in the adjoining soft tissues. Thus, the enamel layer covering the dentin in all ERS patients (except a limited layer of enamel at the dentino-enamel junction) displayed an ultrastructural globular pattern similar to one observed in ectopic mineralization of soft tissue, notably in the gingiva of *Fam20a* knockout mice. XRD analysis confirmed the existence of alterations in crystallinity and composition (vs. sound enamel). XRF identified lower levels of calcium and phosphorus in ERS enamel. Finally, EDS confirmed the reduced amount of calcium in ERS enamel, which appeared similar to dentin.

**Conclusion:** This study suggests that, after an initial normal start to amelogenesis, the bulk of the tissue covering coronal dentin would be formed by different mechanisms based on nano- to micro-nodule aggregation. This evocated ectopic mineralization process is known to intervene in several soft tissues in FAM20A gene mutant.

## Introduction

Hereditary amelogenesis imperfecta (AI) is caused by mutations in genes encoding a number of effectors of amelogenesis such as enamel matrix proteins (amelogenin, ameloblastin, enamelin), peptidases (*MMP20, KLK4*), transcription factors (*DLX3*), and membrane-anchoring polypeptides (laminin 5, collagen 16 laminin; Salido et al., 1992; Barron et al., 2010; Poulter et al., 2014; Seymen et al., 2015; Kim et al., 2016) as well as other polypeptides (ACPT, GPR68, CLDN19; Parry et al., 2016; Seymen et al., 2016; Yamaguti et al., 2017) of unknown function (Prasad et al., 2016). AI can be isolated or syndromic. Recessive *FAM20A* mutations were initially discovered by a whole-exome sequencing of AI patient DNA (O'Sullivan et al., 2011), suggesting an important role of the encoded polypeptide during amelogenesis and in ERS. AI associated with gingival fibromatosis (AIGF MIM#614253; O'Sullivan et al., 2011) or enamel renal syndrome (ERS, MIM#204690; Jaureguiberry et al., 2012; Cabral et al., 2013; Wang et al., 2014; Jaouad et al., 2015; Poulter et al., 2015; Volodarsky et al., 2015) arise due to mutation of one same *FAM20A* gene (Jaureguiberry et al., 2012; Vogel et al., 2012; Chaitanya et al., 2014; de la Dure-Molla et al., 2014; Bhesania et al., 2015). Patients carrying FAM20A mutations present a very distinctive phenotype (de la Dure-Molla et al., 2014): marked hypoplastic AI, important eruption impairment with dental retention and ectopic mineralization in several tissues, including the gingiva, follicular sac, dental pulp, periodontal ligament, and the kidney. Variable semi-lacunar defects at the occlusal edge of permanent upper central incisors have been described. Posterior teeth were reported with a flat cuspid relief wich might be related to either congenital defects or secondary abrasion (Wang et al., 2013). On the other hand, in a number of soft tissues, *FAM20A* loss of function was shown to cause ectopic mineralization (de la Dure-Molla et al., 2014).

The *Fam20a* gene (the name refers to “family with sequence similarity 20”) was initially discovered in mouse hematopoietic cells (Nalbant et al., 2005). Two other members (*Fam20b* and *Fam20c*) were identified by sequence homology, and the proteins they encode (FAM20A, B, and C) are structurally conserved in mice and humans. The most studied member, FAM20C, which was independently identified in odontoblasts as Dentin Matrix Protein 4 (DMP4; Hao et al., 2007), was revealed to be the long-sought Golgi casein kinase (Tagliabracci et al., 2012). This kinase phosphorylates proteins containing canonical Ser-x-Glu/pSer motifs, including a number of matrix phosphoproteins of bone and teeth such as the three major enamel polypeptides, amelogenin, Ameloblastin, enamelin, and osteopontin (Cui et al., 2015; Ma et al., 2016). FAM20B controls glycosaminoglycan assembly by phosphorylating xylose in its elongation common linkage region (Koike et al., 2009). Finally, FAM20A, is considered a pseudokinase due to a mutation within its catalytic site; however, it partners with FAM20C to enhance the latter's Golgi kinase activity. Aberrant tooth phenotypes of null mutant mice reflect the important roles of *Fam20* members in enamel development (Li et al., 2016). Similarly, human *FAM20C* mutations result in AI with hypoplastic enamel (Vogel et al., 2012; Acevedo et al., 2015; Elalaoui et al., 2016), underscoring the importance of the FAM20A-FAM20C interactions in promoting enamel mineralization (Ohyama et al., 2016).

To date, micro CT and scanning electron microscopy of human teeth from patients carrying *FAM20A* mutations revealed crown and root resorption and hypercementosis (Wang et al., 2013, 2014). Increased enamel fragility was suggested, the tissue being quickly worn down by mastication forces after eruption. The chemical composition and ultrastructure of enamel from patients carrying *FAM20A* mutations are still unknown.

This study aimed to characterize the ultrastructure and mineral composition of human enamel of unerupted teeth in a cohort of patients carrying *FAM20A* gene mutations and to compare the findings with those of healthy enamel. We exploited recently developed technological interfaces between physics, chemistry, and biomedical science to analyze biomineralization and map ectopic mineral accretion, as reported previously (Dessombz et al., 2015; Berès et al., 2016). We characterized the enamel from ERS patients using scanning electron microscopy (SEM), X-ray diffraction (XRD), X-ray fluorescence (XRF), and SEM-energy-dispersive spectroscopy (EDS).

## Materials and methods

### Patients recruitment

Twenty-five loss of functions mutations have been reported in the *FAM20A* gene (deletions and base substitutions leading to premature stop codon). Patients carrying FAM20 gene mutations (*n* = 6) is a cohort recruited in the Reference Center of rare dental disease in Paris (Rothschild hospital) being part of previously published cases of ERS (Jaureguiberry et al., 2012). Diagnosis of ERS was based on clinical and radiological features (enamel hypoplasia, eruption impairment, and pulp mineralization) and *FAM20A* mutations as previously published (de la Dure-Molla et al., 2014). Affected individuals and controls were recruited following informed consent in accordance with the principles outlined in the declaration of Helsinki. According to the French law, the samples were considered as operating waste and used under patient informed consent. Erupted (*n* = 3) and unerupted teeth (*n* = 9) from 3 differents ERS patients and permanent teeth from healthy subjects (*n* = 6) were collected after their extraction, based on the treatment plan.

### Sample preparation

Teeth were rinsed with PBS (Invitrogen, Carlsbad, CA) and fixed in 4% paraformaldehyde (Electron Microscopy Sciences, Hatfield, PA). Samples were then dehydrated in alcohol, embedded into light-cured methacrylate resin (Technovit 7200 VLC; Heraeus Kulzer, Hanau, Germany) and cut into 100–150 μm thick slices using a low-speed diamond saw under irrigation (Isomet Low Speed Cutter; Buehler, Dusseldorf, Germany). Finally, the samples were polished using graded grit polisher disks.

### Scanning electron microscopy

Sections were sputter-coated with a 6-nm layer of platinum (SC7640 sputter coater; Quorum Technologies, Guelph, ON, Canada). A SUPRA 40 Scanning Electron Microscope (SEM; Carl Zeiss, Oberkochen, Germany) was used to observe the microstructure of the teeth. This field-effect gun microscope operates at 0.5–30 kV. Observations of sectioned samples were made by using an Everhart-Thornley Secondary Electron (SE) detector at 20 keV and with a backscattered electrons (BSE) detector at 20 keV.

### X-ray diffraction

Chemical phase and crystallinity of the enamel mineral were evaluated by X-ray diffraction (XRD). Experiments were carried out with a Molybdenum rotating anode X-ray generator (Rigaku RU-H2R; Rigaku, Tokyo, Japan) coupled with multilayer W/Si optics (Xenocs, Grenoble, France) delivering a focalized and monochromated (λ = 0.711 Å) X-ray beam of 800 μm × 1 mm onto the sample. X-ray images were recorded with a MAR345 (marXperts, Hamburg, Germany) detector placed 150 mm from the sample. The acquisition time for each measurement was 30 min. Diffraction diagrams were obtained by processing radial intensity integration of each image with in-house software. Then, the positions of the diffraction peaks were compared with reference files from the International Center for Diffraction Data (ICDD).

### X-ray fluorescence

X-ray fluorescence (XRF) experiments were carried out with Molybdenum rotating anode X-ray generator (Rigaku RU200) coupled with multilayer W/Si optics (Xenocs) delivering a focalized and monochromated (λ = 0.711 Å) X-ray beam of 150 × 150 μm. Fluorescence spectra were measured with an energy-dispersive detector (SDD detector, Ketek), with a time acquisition of 240 min. XRF analysis was performed with PyMca software (Solé et al., 2007).

### Energy dispersive spectroscopy

A SUPRA 55 SEM (Carl Zeiss, Oberkochen, Germany) equipped with an energy-dispersive X-ray spectrometer (Bruker SDD detector) was used to perform the observations and chemical analyses. This field-effect “gun” microscope (FE-SEM) operates at 0.5–30 kV with an energy of 25 kV. High-resolution observations were obtained by 2 secondary electron detectors: an in-lens SE detector and an Everhart-Thornley SE detector. The acquisition mode only permits qualitative analysis because no control sample was used.

## Results

### Scanning electron microscopic analysis reveals dramatic enamel hypoplasia in ERS

**Figure 1:**
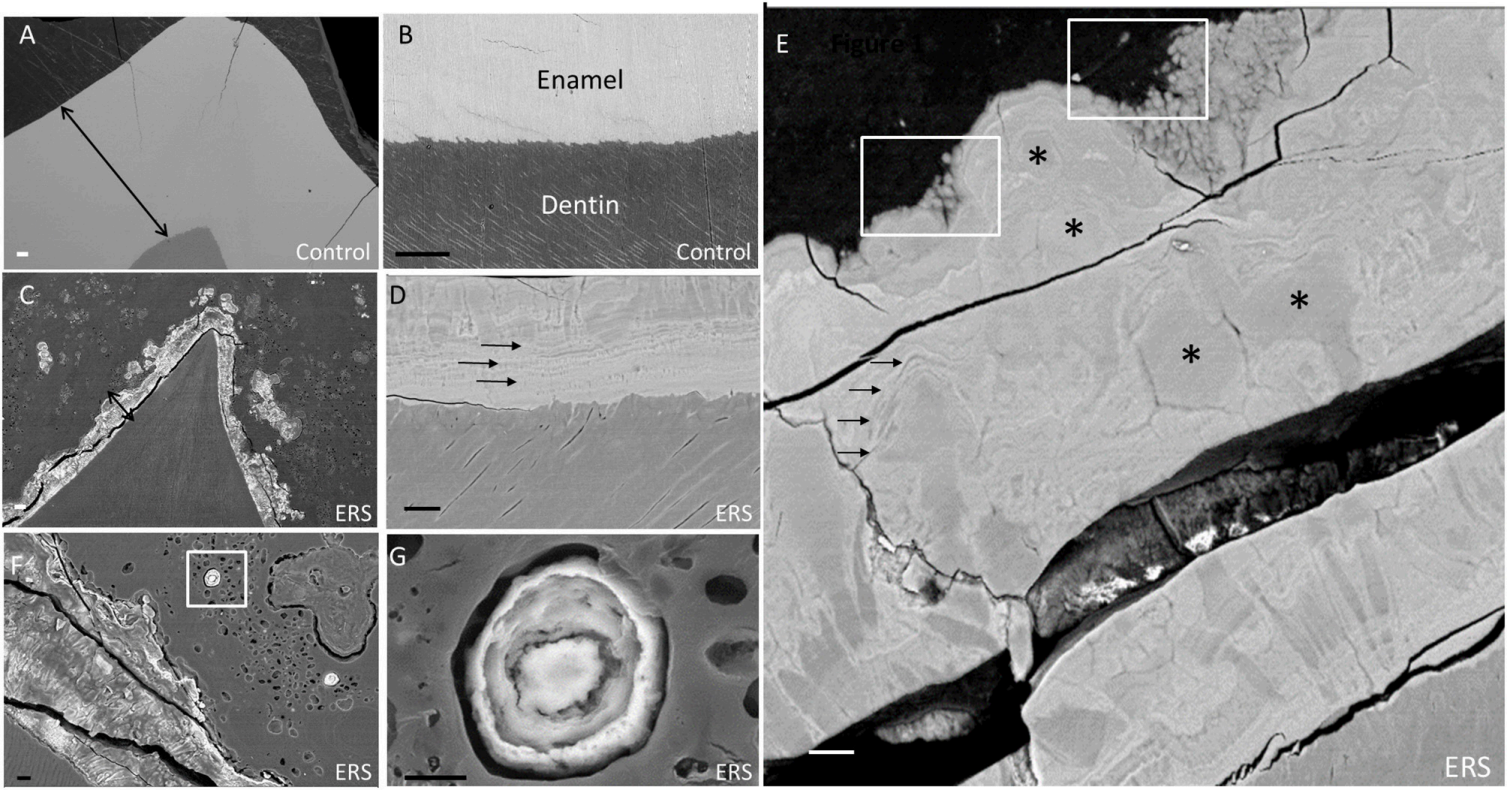
**SEM images of enamel from erupted teeth of a healthy subject and unerupted teeth from an ERS patient (mutated for *FAM20A*). (A)** Sound enamel of a healthy tooth. **(B)** Sound dentino-enamel junction. Double arrows underlining a reference and identical scale in **(A,C)**. **(C)** Enamel from an ERS patient. **(D)** Dentino-enamel junction from an ERS patient. Single arrows highlighting the presence of an enamel organized in different layers. **(E)** Enamel outer layer from an ERS patient. Asterisks and white boxes show the presence of micro- and nano-nodules in enamel layers and arrows highlighting the presence of enamel organized in different layers. **(F)** Enamel organ from an ERS patient. Nodule calcification in box is represented at higher magnification in G. **(G)** Higher magnification of nodule calcification in the enamel organ shown in **(F)**. Scale bar: **(A–C)**, 100 μm; **(D–G)**, 20 μm.

SEM images (Figure 1) were obtained using the BSE mode to analyze chemical contrast and the SE mode to visualize morphology. Healthy enamel displayed a normal thickness and a high level of mineralization in comparison to dentin (Figure 1A). Moreover, at the Dentino-Enamel Junction (DEJ), using BSE mode, sound enamel showed a uniform electron density (Figure 1B), suggesting the presence of a homogeneous chemical phase.

All ERS erupted teeth presented enamel breakdown (data non-shown) suggesting their decreased mechanical resistance as described by other studies (Wang et al., 2013). Representative features for enamel and ectopic mineral present in all ERS unerupted teeth are shown in Figures 1C–G. There, the enamel displayed a severe reduction in thickness It should be noted that the images in panels A and C are at the same magnification, thus highlighting the dramatic native enamel hypoplasia in ERS. Samples appeared to be fractured, suggesting their low resistance (Figures 1C–E), in contrast with controls (Figures 1A,B).

At the DEJ, enamel from an ERS patient (Figure 1D) exhibited layers of alternating dark and light areas (arrows), signifying differences in electron density (in BSE mode). This finding raised three non-exclusive hypotheses for the observed differences between healthy and ERS enamel: (1) differences in chemical phase, (2) differences in qualitative composition, and/or (3) differences in mineral density, which we investigated in turn in the subsections below. Indeed, BSE electrons result from elastic interactions with the nuclei of atoms. The higher the atomic number (Z), the higher the probability of an elastic interaction, and the brighter the contrast.

Fused nodules of varying sizes were found throughout the enamel thickness (Figures 1C,E,F,G). Most of the enamel layers from ERS patients were composed of micro- and nano-nodules (Figure 1E, asterisks—white boxes). In the major part of enamel, enamel prisms tended to be restrained to the most inner zones (Figures 1C,F). Nodules ranged from 1 to 50 μm in diameter, separated poorly electron dense frontiers and embedded in more or less regular lamellae (arrows; Figure 1E). Nanonodules with a concentric organization of varying sizes were present at the surface of ERS enamel and also within the enamel organ bordering the enamel [Figure 1F (white box) with enlargement in Figure 1G].

### X-ray diffraction reveals small and disoriented crystallites in ERS enamel

**Figure 2:**
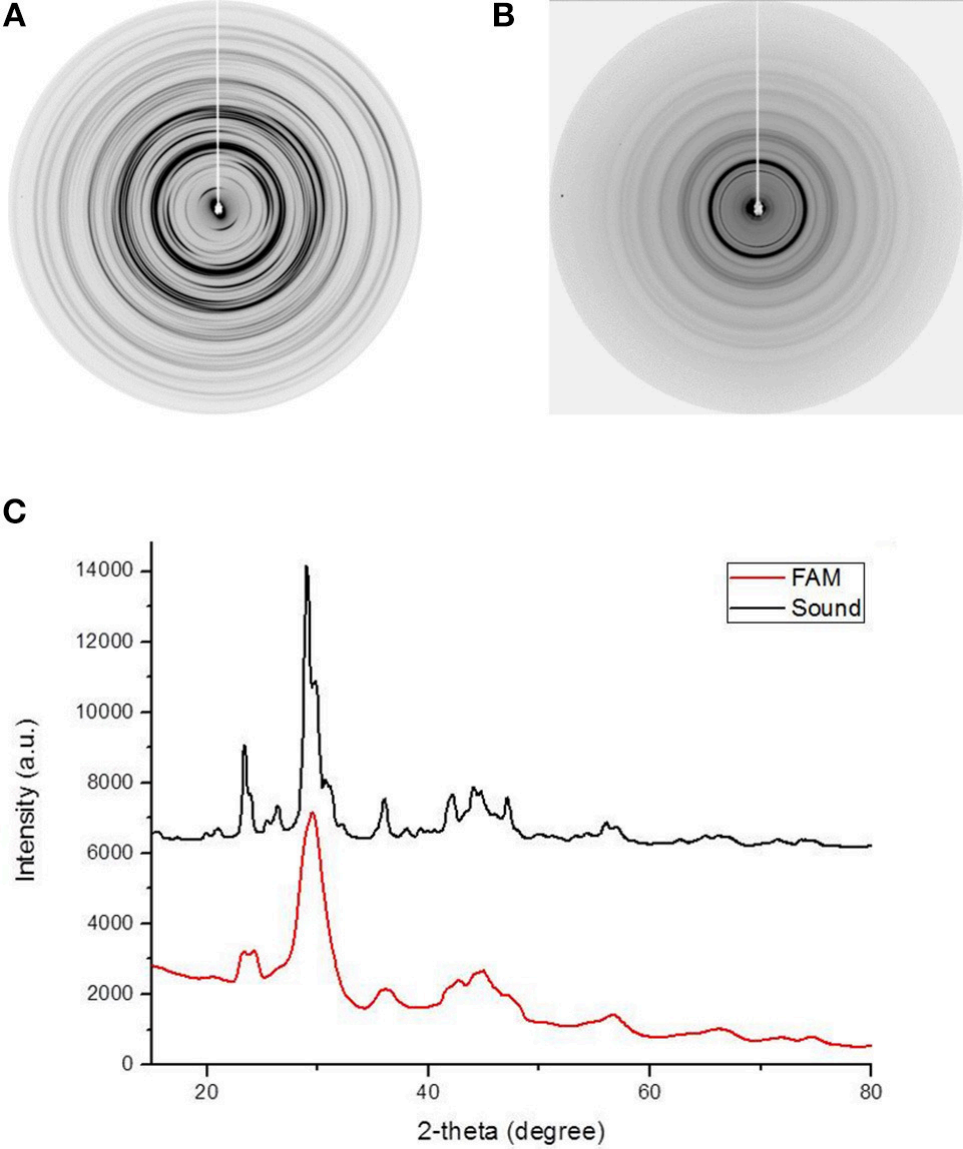
**Two-dimensional XRD patterns produced by (A)** healthy enamel and **(B)** enamel from an ERS patient. **(C)** Diffraction diagram obtained by radial intensity integration of the diffraction images in **(A,B)**.

XRD was used to determine if differences in the crystalline phase exist between sound enamel (Figure 2A) and ERS enamel (Figure 2B). The general features of both two-dimensional XRD patterns were similar, with the same number and position of diffraction rings. However, several differences were observed: first, the ERS enamel displayed continuous powder-like diffraction rings, indicating a loss of texture compared to sound enamel. This corresponded to an isotropic crystalline orientation in ERS enamel. Conversely, in sound enamel, the ring patterns were textured according to the anisotropic orientation of the prisms. Radial integration profiles (Figure 2C) provided diffraction diagrams as a function of 2θ diffraction Bragg angles. The diffraction diagrams from both enamels showed similar characteristics, with significant peaks located at identical 2θ angles; however, the diffraction diagram of ERS enamel displayed broader and overlapping peaks. These results indicate the same crystalline phase, but with smaller and disoriented crystallites in ERS enamel. Phase identification confirmed the presence of carbonated apatite (ICDD 09-432) in all tested samples, which is a normal constituent of healthy enamel.

### X-ray fluorescence analysis indicates a calcium-phosphate mineral phase in ERS enamel

**Figure 3:**
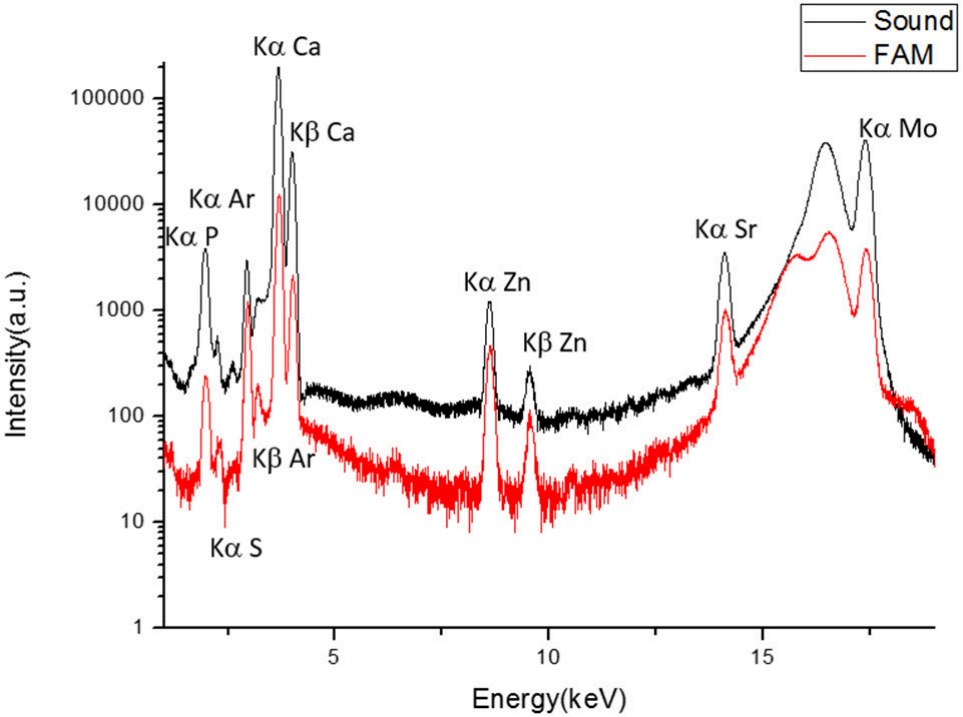
**XRF spectra of sound enamel and enamel from an ERS patient**. Contributions of P (Kα = 2.014 keV), S (Kα = 2.307 keV), atmospheric Ar (Kα = 2.958 keV), Ca (Kα = 3.692 keV, Kβ = 4.012 keV), Zn (Kα = 8.638 keV, Kβ = 9.572 keV), Sr (Kα = 14.165 keV), and Mo (17.48 keV). The peaks at 16.534 and 17.48 keV are Compton scattering and irradiation, respectively.

XRF was used to analyze chemical composition in healthy and ERS enamel. The XRF spectra identified phosphorus, argon, calcium, zinc, and strontium in both enamel samples (Figure 3). The presence of Ca and P further supported that the mineral phase is a calcium phosphate, in accordance with the XRD results shown in Figure 2. Presence of Zn and Sr, two trace elements, is related to calcium substitution. Ar is present in the atmosphere along the path of the incident X-ray beam.

### Energy dispersive spectroscopy reveals reduced mineralization in ERS enamel

**Figure 4:**
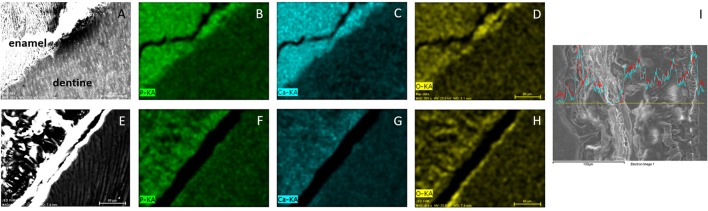
**EDS mapping of enamel and dentine from healthy donors (A–D)** and from FAM20A patient **(E–H)**. Images are first viewed in SEM **(A,E)**. Contributions of P **(B,F)**, Ca **(C,G)** and O **(D,H)** are then analyzed. **(I)** Enamel section with variation of Ca (blue) and P (red) across the section. Scale bar: 80 μm, except for **(I)** 100 μm.

EDS was used to analyze mineral density. The results are shown in Figure 4 and present the EDS elemental mapping (Spectral imaging mode) of phosphorus, calcium and oxygen in sound enamel (Figures 4A–D) and ERS enamel (Figures 4E–H). In sound enamel, P (Figure 4B) and Ca (Figure 4C) were present in higher amounts in comparison to dentin. This result underlines the high degree of mineralization in sound enamel. In contrast, there was no such difference between the enamel and dentin from an ERS patient (Figures 4E–H), demonstrating the reduction of enamel mineralization in ERS. Furthermore, spectra of Ca and P in ERS patient enamel showed significant variations through the enamel (Figure 4I).

## Discussion

Amelogenesis is a complex process which results in the secretion of an acellular matrix by ameloblasts. In the first secretion stage, ameloblasts export enamel matrix proteins (EMPs) required for the deposition of the different enamel layers (Lignon et al., 2015). After the deposition of a first internal aprismatic enamel, the ameloblasts produce internal prismatic enamel. Finally, external prismatic and outer aprismatic enamels are deposited. The organized patterns of crystal directions in these specific aprismatic and prismatic layers confer on enamel its anisotropic structure (Simmons et al., 2011; Al-Jawad et al., 2012). Then after, ameloblasts produce extracellular proteases such as metalloproteinase (MMP20) or kallikrein 4 (KLK4) which cleave EMPs. During the maturation, their proteolysis and removal from the matrix allow the lateral growth of the crystal. The constitutive components of intraprismatic and interprismatic enamel are hexagonal hydroxyapatite crystals (Lignon et al., 2015). Several EMPs play a germinal role in enamel formation, mainly the amelogenins (AMEL) which regulate mineralization by controlling enamel thickness. Their supramolecular organization sets up the crystal elongation axis and the prism pattern. Ameloblastin (AMBN) is located between intra- and inter-prismatic enamel and establishes the supra-crystalline organization of enamel. Enamelin (ENAM) is required for crystal growth and elongation. FAM20A is produced by ameloblasts (Wang et al., 2014), and by interacting with the FAM20C kinase would help phosphorylate these EMPs (Vogel et al., 2012).

Mutation of EMP genes leads to AI, which may be hypoplastic, hypomature, or hypomineralized (Lignon et al., 2015). Autosomal dominant AI comprises 46–67% of all AI. The most frequently affected genes are *ENAM* and *FAM83H* (involved in ameloblast differentiation). In 2014, Poulter et al. identified a mutation in *AMBN* which also gives rise to AI (Poulter et al., 2014). AI associated with *AMELX* gene mutations (located on the X chromosome) is involved in 5% of cases, all of which feature a striated enamel appearance. Autosomal recessive forms involving six genes, but mainly kalikrein 4 (*KLK4*) and metalloprotease-20 (*MMP20*), are the scarcest. In these latter forms of AI, enamel exhibits a normal thickness but is more fragile and frequently worn. More recently, *FAM20A* gene mutations have been identified in hypoplastic AI associated with gingival fibromatosis (O'Sullivan et al., 2011) and with nephrocalcinosis (Jaureguiberry et al., 2012; Wang et al., 2013). The present data support and extend previous studies on teeth from ERS patients (Wang et al., 2013, 2014) by furnishing details of the ultrastructure of the tissue covering the dentin as well as its physicochemical properties.

XRD diffractograms proved the presence of the same crystalline phase in sound enamel and ERS enamel. Differences were, however, observed in the width and intensity of the diffraction rings, suggesting some morphological and structural changes. Concerning diffractograms, the broader the peaks, the smaller the diffracting crystallites. ERS enamel therefore displays smaller size crystals. Moreover, the powder-like diffractograms indicates a loss of structural organization between the crystallites, in contrast with the prismatic organization in sound enamel. In this study, it should be kept in mind that this technique presented some limitations due to the resolution. The major limitation of a smaller-diameter beam is the reduction in luminous flux. A superior alternative would be to use a synchrotron beam, which provides better resolution.

As the crystalline phase was found to be the same in sound and ERS enamel, we then investigated, by XRF, possible differences in the presence of trace elements. The XRF data revealed a similar elemental composition for both samples, including the presence of Ca and P. The Ar detected originated from gas in the atmosphere. Moreover, two trace elements were detected, Zn and Sr, which correspond to Ca substitution. Indeed, Zn is an essential trace element for living organisms. It takes part in many aspects of metabolism and may be inserted into hydroxyapatite. In apatite, Zn may substitute for up to 5% of Ca (Bazin et al., 2009). This substitution does not induce modification in lattice parameters (Ren et al., 2009). Moreover, Zn may also be a marker of inflammation (Dessombz et al., 2013). As observed here, Sr is also commonly found in biological apatite as a substitute for Ca (Schroeder et al., 1972). In long bones, Sr promotes biomineralization and is used as a preventive in osteoporosis (Bone et al., 2013; Bazin et al., 2014). Other research groups have applied XRF to detect other trace elements in tooth enamel (Oprea et al., 2009; Zimmerman et al., 2015), also resulting from Ca substitution. The other elements we identified were As, Ti, Li, Be, Mg, Al, Mn, Fe, Cu, Zr, Sn, Au, Hg, Pb, Ac. Additionally, phosphate and hydroxyl groups may be substituted by F and Cl (Oprea et al., 2009; Zimmerman et al., 2015), which was also what we found here.

On the other hand, XRF did reveal differences in the intensity of elemental composition between sound and ERS enamel. The increased intensity observed for trace elements in ERS enamel was probably due to the enamel's reduced thickness and exchanges with the environment. Indeed, in 1989, Frank et al. showed by ED-XRF a higher concentration of Zn and Sr in outer enamel vs. inner enamel (Frank et al., 1989).

Finally, we used EDS to analyze mineral proportion in tissue. Our results showed higher calcium concentration in enamel compared with dentin in healthy teeth, underlining the important proportion of mineral in this calcified tissues. In contrast, ERS enamel and dentin showed similar levels of mineralization, indicating a weakly mineralized enamel. The deficiency left by the lower Ca level in ERS enamel is probably filled by organic species, like proteins. The XRF and EDS methods are complementary owing to their different detection thresholds. Indeed, low-Z elements like oxygen cannot be detected by XRF, whereas the concentrations of the trace elements Sr and Zn were probably too low to be detected by EDS.

The present study evidenced alterations in patients carrying *FAM20A* mutations, with major disturbances in enamel morphogenesis and biomineralization. SEM and physicochemical methods highlighted specific mineral morphology or composition, suggesting some pathological mechanisms and revealing conditions of the mineral formation. In ERS enamel, we observed a prismatic-like structure exclusively in the most internal enamel, suggesting normal initiation of the amelogenesis process. In contrast, in the rest of the enamel, our SEM data highlight the heterogeneity of ERS enamel structure. SEM imaging revealed nano- to micro-meter nodules, increasing in size from the outer to the inner zones. This would suggest that, in contrast to normal enamel in which enamel crystals continuously elongate throughout the enamel thickness, independent nanonodules would be produced which fuse to form micronodules. This process, also observed in the adjoining soft tissues shows some similarities with ectopic mineralization described in the gingiva (Vogel et al., 2012). The produced tissue remains highly heterogeneous and the spatial distribution of atomic mineralization's markers (Ca and P) is strongly disturbed.

The role of FAM20A in amelogenesis may be indirect, as FAM20A binds FAM20C kinase to promote phosphorylation of secreted polypeptides *in vitro* (Ohyama et al., 2016). Indeed, the three major enamel matrix proteins (AMEL, AMBN, and ENAM) contain the amino-acid motif enabling phosphorylation by FAM20C. Furthermore, several studies have demonstrated the essential role of EMP phosphorylation during amelogenesis. First, in 2010, Chan et al. showed that, in humans, lack of ENAM phosphorylation gives rise to AI (Chan et al., 2010); in cases where only one allele was affected, minor pitting or enamel hypoplasia was observed. In patients where both alleles were affected, severe enamel malformations were observed, with little or no mineralize material covering the dentin. In 2016, Ma et al., using transgenic mice with a phosphorylation-defective AMBN polypeptide, found severe enamel defects such as hypoplasia, severely disturbed enamel rods and interrod structure, and enamel matrix invading the ameloblast layer (Ma et al., 2016). Thus, it may be hypothesized that FAM20A loss of function would result in reduced phosphorylation of EMPs, thus disrupting amelogenesis beyond the first stages of inner enamel deposition, and leading to a poorly mineralized matrix.

A second physiological role for FAM20A would be to inhibit ectopic mineralization. Indeed, *FAM20A* mutations in humans (this report) or *Fam20A* deficiency in mouse knockout (KO) models are associated with ectopic mineralization in the gingiva and enamel organ, suggesting that some mineralization inhibitors may be additional FAM20A targets. Among many potential candidates, fetuin may be one such putative inhibitor. Indeed, fetuin deficiency results in an ectopic calcification phenotype, resembling that in *Fam20a* KO mice (Schäfer et al., 2003; Vogel et al., 2012; Ohyama et al., 2016).

In order to define more definitively this sequence of enamel formation and/or ectopic mineral deposition in the context of *FAM20A* gene mutations, an ultrastructural analysis of enamel, of the produced EMPs and extracellular peptides in the available animal models would be of great interest.

## Author contributions

Conceived and designed the experiments: GL, FB, AD, and AB. Performed the experiments: GL, FB, AD, SR, and RW. Analyzed the data: GL, FB, AB, and AD. Wrote the paper: GL, FB, RK, and AD. Teeth were collected by MQ, MD and AN.

### Conflict of interest statement

The authors declare that the research was conducted in the absence of any commercial or financial relationships that could be construed as a potential conflict of interest.
